# Does Government Intervention Ensure Food Safety? Evidence from China

**DOI:** 10.3390/ijerph18073645

**Published:** 2021-03-31

**Authors:** Hongfeng Zhang, Chengyun Sun, Lu Huang, Hongyun Si

**Affiliations:** 1 School of Public Administration and Policy, Shandong University of Finance and Economics, Jinan 250014, China; zhanghongfeng@sdufe.edu.cn; 2 School of Economics, Shandong University of Finance and Economics, Jinan 250014, China; chyuns2001@163.com (C.S.); huanglu9609@163.com (L.H.)

**Keywords:** government intervention, food safety, spatial econometrics, local government competition, China

## Abstract

Food safety is related to public health, social welfare, and human survival, all of which are important and pressing areas of concern all over the world. The government plays an increasingly important role in the supervision of food safety. The role of the government, however, is also controversial. Using provincial panel data of China from 2005 to 2015, the present study intends to shed light on the associations between government intervention and food safety performance under two scenarios of local government—competition and noncompetition. This will be accomplished through an exploratory spatial data analysis and a spatial econometric model. The results reveal negative associations between food safety performance and government intervention without considering local government competition. As was also observed, government intervention not only inhibits the improvement of food safety in the region, but also has a negative spatial spillover effect on food safety in neighboring provinces. This is the result after considering government competition, thus, showing the competitive strategic interaction of the “race to the bottom”. Further analysis reveals that, if geographically similar regions are selected as reference objects, the food safety performance of each province will have a stronger tendency to compete for the better. If regions with similar economic development levels are selected as reference objects, food safety performance will have a stronger tendency to compete for the worse. This work provides new evidence for the relationships between government intervention and food safety, and, also, proposes some insightful implications for policymakers for governing food safety.

## 1. Introduction

In the past few years, global food incidents have occurred frequently [1,2], and the problem of food waste is serious [3]. These issues have aroused widespread concern all over the world. Food incidents in various countries have shaken consumer confidence in food safety; the acceleration of economic globalization has also made the food supply chain more complicated [4]. Guaranteeing global food safety will require the joint efforts of governments and relevant subjects.

In 2019, China ranked 35th out of 113 countries in food security, according to the Global Food Security Index (GFSI) [5], and there is still much room for improvement. In China, the measures being implemented to improve people’s living standards mainly focus on the environment, water, and personal hygiene, while food safety has been neglected [6]. In the past, food safety issues, such as those associated with gutter oil and clenbuterol, not only created challenging issues with regard to public hygiene, nutrition, and health in China; they also caused a series of social and ethical problems, such as the decline of social trust [7,8]. Therefore, China has begun a comprehensive reform of the country’s food safety management system [9]. In October of 2016, the Chinese government published “the Plan of Health China 2030”, which aims to improve the health level of Chinese people. Food safety is one of the three key links of the Health China strategy.

Although some countries, including China, have taken measures to strengthen the supervision of food safety, weak links in the implementation of these measures still exist [10]. In the market, information asymmetry has led to a serious industry trust crisis. Moreover, the gap between the supply and demand of public products is large, and market failures, such as the prominent externality of food safety issues, frequently occur. This situation creates the necessity for government intervention. However, government intervention policies have traditionally also been troubled by government failures, which, in turn, are caused by the government’s own defects [11]. Therefore, the scope, intensity and measures of government intervention have become key factors in determining food safety situations.

In the past few decades, many scholars have attempted to reveal the effects of government intervention on economic and social development. Government intervention measures mainly include the direct control of enterprises, as well as indirect economic regulation, such as tax subsidies, financial regulation, and labor mobility restrictions. Existing government intervention studies mostly focus on the impact of government intervention on various aspects of economic development, and different opinions have been expressed. Some scholars hold that government intervention is effective in achieving the stated goal. For example, Wang revealed the impact of government intervention on innovation performance in Hong Kong and Singapore [12]. The study found that appropriate government intervention is effective in highlighting the value of science and technology, and in expanding the scope of innovation. Fang et al. found, through dynamic simulation and data fitting, that government intervention has played a significant role in suppressing the spread of the coronavirus disease 2019 (COVID-19) [13]. Nevertheless, some scholars suggest that there are many drawbacks in the government intervention process intended to promote economic development. For instance, Chen et al. found that government intervention imposed on state-owned enterprises can distort investment behavior and damage investment efficiency [14]. Zhang et al. found that the government’s excessive pursuit of economic performance has led to overinvestment and overcapacity problems [15]. In addition, local governments in China have promoted economic growth by lowering the price of industrial land and raising the price of commercial and residential land. These practices have led to serious land price distortion and resource mismatches [16], thus further reducing the use efficiency of industrial land [17].

The history of human social progress has been accompanied by the solution and development of food safety problems. Consequently, numerous relevant scholars have also launched a significant number of discussions related to food safety. Xiong et al. evaluated the performance of the food safety management system (FSMS) of pork slaughter plants in China [18]. The study found that factors such as company size, location, and market all play an important role. Han et al. uncovered that people with different sociodemographic characteristics have different perceptions of food safety risks [8]. Lundén et al. found that food safety performance in China is closely related to the degree of economic development and urbanization of those regions [19]. In addition, Lu et al., Zhang et al., Toth et al., and Carvalho, respectively, studied the effects of soil and water pollution, pesticides, and fertilizers on food safety [20,21,22,23]. Hernández-Rubio et al. took Spain and France as examples to explore the factors that influence the safety levels of vegetable and fruit supply chains [24]. Liao et al. explored the impact of food recalls on consumer behavior and food safety [25].

The question must then be asked, has government intervention really improved food safety performance (FSP)? Some studies have shown that government intervention has strengthened the motivation of food production companies to provide safer food. Thus, government intervention has played an increasingly important role in the food supply system [26]. Zhang et al. found that the control of food safety not only depends on the companies and partners in food supply chains, but also on the continuous improvement of the government’s supervision system [9]. Governments mainly intervene and supervise food safety by formulating policies and regulations, while social welfare is improved through the optimization of resource allocations [2]. Ortega et al. found that Chinese consumers are more inclined to buy government-certified products [27]. The study therefore suggested that the Chinese government should directly participate in the construction of the food safety system. Conversely, some scholars are skeptical about the role of government intervention. Jia and Jukes pointed out that, although China’s food regulatory system has made significant improvements in terms of overall framework design, the food regulatory system is still insufficient in terms of standard formulation, law enforcement, and information exchange [28]. Chu found that China’s food safety regulations have a significant deterrent effect on the export food sector. However, the regulations’ effect on the domestic food market is relatively limited [29].

The existing research on government intervention and food safety is relatively rich, but there are still some deficiencies. One is the lack of empirical research. Owing to the lack of data on food safety costs, benefits, and foodborne diseases, the number of empirical studies containing such information is limited [26]. There is also a lack of empirical research that considers the spatial spillover effect. Secondly, existing research rarely considers the association between government intervention and food safety. In addition, research that considers the factors of local government competition is even scarcer. The purpose of the present study is to investigate the direct and spatial effects of government intervention on food safety performance. In order to achieve this goal, this research attempts to use a spatial Durbin model (SDM) and an asymmetric response model to explore the association between government intervention and food safety performance, based on provincial panel data from China. This research, for the first time, empirically explores the association between Chinese government intervention and food safety performance. The spatial spillover effect of government intervention is revealed, and comparisons are made of the results under the two situations, i.e., whether or not local government competition is considered. This study therefore provides new evidence regarding the association between government intervention and food safety. Ultimately, this study proposed some targeted policy recommendations related to the association between Chinese government intervention and food safety.

## 2. Materials and Methods

### 2.1. Model Description

**Figure 1:**
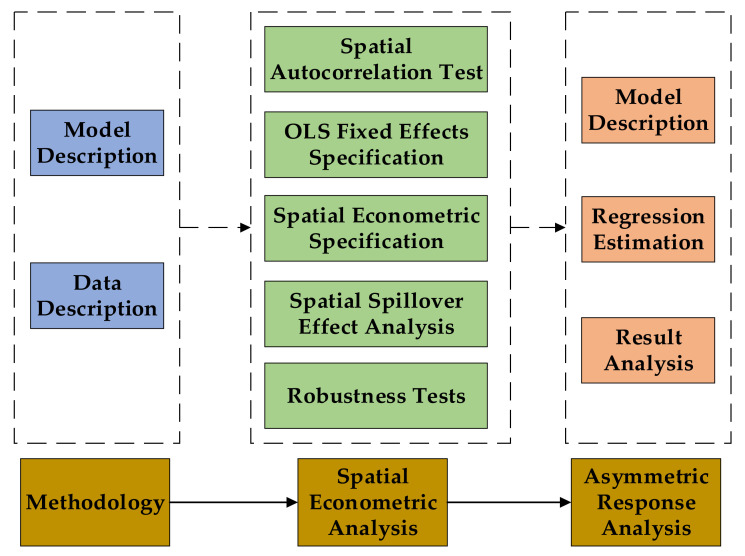
Research framework and process.

In this study, the model was set up and the data was described firstly. Then, the spatial econometric analysis and the asymmetric response analysis were carried out. For the empirical analysis, the external commands in Stata software were used for model building and estimating. In order to better show the research process of this study, the research framework is presented in Figure 1.

This research estimated the associations between government intervention and food safety performance under two situations, namely when local government competition is considered and when that competition is not considered. The ordinary least squares (OLS) method was used to estimate regression. The Hausman test was used for fixed effect (FE) and random effect (RE). The test results were significant. Therefore, this paper used a two-way fixed effect model for estimation. The regression model for estimating was as follows:

(1) $$Perform_{i,t}=\alpha +\beta_{1}Govern_{i,t}+\sum \beta_{j}X_{i,t}+\gamma_{t}+\mu_{i}+\varepsilon_{i,t}$$

where the dependent variable ($Perform_{i,t}$) is the measurement of the food safety performance in province *i* at year *t*; the key explanatory variable ($Govern_{i,t}$) is the degree of government intervention in province *i* at year *t*; $X_{i,t}$ indicates a series of control variables, including the economic development level, degree of openness, degree of marketization, industrial structure, population growth rate, technological innovation, urbanization level and education level; $\gamma_{t}$ represents year fixed effects, capturing all yearly factors common to all provinces (such as economic cycle, monetary policy, etc.); $\mu_{i}$ represents province fixed effects, which capture all provinces’ time-invariant characteristics (such as geographic features, natural endowment, etc.); $\varepsilon_{i,t}$ is the error term. The specific variable setting is described in detail later in this paper. In the above model, $\beta_{1}$ is the core estimation parameter, which represents the associations between government intervention and food safety performance. If $\beta_{1}$ is positive, this means that increased government intervention is beneficial to the improvement of food safety.

Due to development needs and performance evaluations, local governments will inevitably compete with each other. Government intervention—achieved by influencing market supervision—has become an important factor in the strategic food safety competition between local governments. In this research, the spatial relationship of each region was considered by the spatial weight matrix to test the associations between government intervention and food safety when considering local government competition. Since government intervention was an endogenous variable, this article drew on Lesage and Fischer [30] and introduced the SDM, which could effectively solve the endogenous problem. The estimation model was constructed as follows:

(2) $$Perform_{i,t}=\alpha +\rho WPerform_{i,t}+\beta_{1}Govern_{i,t}+\sum \beta_{j}X_{i,t}+W\delta_{1}Govern_{i,t}+\sum W\delta_{j}X_{i,t}+\mu_{i}+\varepsilon_{i,t}$$

where $Perform_{i,t}$, $Govern_{i,t}$ and $X_{i,t}$ are the same as in Equation (1); ρ is the spatial autoregressive coefficient of the dependent variable; $\beta_{1}$ is the effect of government intervention on food safety performance; $\delta_{1}$ is the spatial spillover effect of government intervention; $\beta_{j}$ is the effect of other control variables on food safety performance; $\delta_{j}$ is the spatial spillover effect of other control variables; $\mu_{i}$ is the cross-sectional intercept term which donates the spatial fixed effects; $\varepsilon_{i,t}$ is the error term without spatial autocorrelation; and W is the spatial weight matrix. Part of the data from Tibet Province, China, is missing, so, the sample utilized for the empirical analysis was comprised of the remaining 30 provinces in mainland China. Then, a 30 × 30 spatial weight matrix was constructed. Local government competition is based more on geographic distance. Therefore, this research used the binary adjacency weight matrix (Ad-weight) and geographic distance weight matrix (Geo-weight). Here, Ad-weight meant that, when two provinces were geographically adjacent to each other, a spatial correlation existed between them, and the value was 1. Otherwise, the value was 0. Then, the elements of the weight matrix were standardized. In order to avoid the islanding effect, Hainan Province was set to be adjacent to Guangdong and Guangxi Province; Geo-weight took into account the real geographical distance of each province. In addition, the weight matrix was set according to the reciprocal of the center distance of each provincial capital city, in order to explore the differential spatial spillover effects of different provinces in similar regions.

One can judge the strategy interaction mode of local government competition according to the sign and size of $\beta_{1}$ and $\delta_{1}$. If $\beta_{1}$ was significantly positive, government intervention could promote food safety performance. At this time, if $\delta_{1}$ was positive, this meant that neighboring areas have taken action to strengthen government intervention, thus forming yardstick competition. There was also a positive spatial spillover of government intervention. If $\delta_{1}$ was negative, this meant that neighboring areas have reduced the level of government intervention and have formed differentiated competition. Here, the government intervention had a negative spatial spillover. If $\beta_{1}$ was significantly negative, government intervention inhibited the improvement of food safety. At this time, if $\delta_{1}$ was positive, this meant that there was a differentiation competition. If $\delta_{1}$ was negative, however, this meant that there was a “race to the bottom” competition.

### 2.2. Data

This paper focused on the associations between government intervention and food safety; the spatial spillover effect of government intervention and robustness of the results were also analyzed in detail. In addition, there are many factors that affect regional food safety, so, other control variables were introduced. The specific variable selection and calculation method are shown in Table 1.

#### 2.2.1. Dependent Variable

The key dependent variable of this study was the food safety performance of each province. Owing to the limitation of data availability pertaining to positive indicators of food safety, this article, referring to Zhang et al., used the reciprocal of the number of food safety incidents in each province, every year, as the proxy variable of food safety performance [31]. According to the World Trade Organization (WTO), a public health event is defined as a food safety incident that affects human health. There were two types of food safety incidents in this study. One was foodborne disease, food contamination, and other events that originate from food, harm to human health or may cause harm. Food contamination includes excessive use of additives, pesticide residues, pathogenic microorganism contamination, and radioactive contamination. The second type was the events reported by social public opinion that had a negative impact on consumers’ food safety consumption psychology. The relevant data came from the “Introduction to the 2016 China Development Report on Food Safety”, which was jointly released by Jiangnan University, Qufu Normal University, China Food Safety News, and the Research Center of China Food Safety Public Sentiment. Since the data of this indicator were published up to 2015, the sample period selected for this study was from 2005 to 2015.

#### 2.2.2. Key Explanatory Variable

The explanatory variable of this research was government intervention. This research was limited by the availability of data, as government intervention indicators cannot be obtained according to the corresponding specific policies; such indicators are also usually measured by constructing proxy variables [14,16]. Therefore, this research referred to the practice of Shi and Shen, and used the ratio of the relevant local government’s financial expenditure on food safety supervision to regional GDP as the means to measure government intervention [32]. The government’s fiscal expenditure on food safety included food safety supervision expenditure, food quality inspection expenditure, food enterprise subsidy expenditure, food safety city constructing expenditure, and food safety knowledge popularization expenditure. Since the data pertaining to the Chinese government’s financial expenditure on food safety supervision were not available, this study took the general budget expenditure of the local government, multiplied by the ratio of national food industry output value and GDP in each year, as the proxy variable of the local government’s financial expenditure on food safety supervision. The data were taken from the National Bureau of Statistics of China, the China Statistical Yearbook, and the China Food Industry Yearbook.

#### 2.2.3. Control Variable

In order to control the impact of other factors on food safety, this research selected a series of control variables on the basis of combing relevant extant literature. Previous studies have found that both economic development level and urbanization level are important factors affecting food safety [31]. Consequently, this study used the ratio of the real GDP after deflating, based on 2005, to the total population at the end of the year, to express the economic development level. Furthermore, the ratio of the urban population to the total population at the end of the year was used to express urbanization level. With the continuous advancement of economic globalization, China’s opening up and international trade have had an important impact on food supply chain security. Therefore, this article used the ratio of the actual utilization of foreign direct investment (FDI) to GDP in a region to indicate the openness degree [33]. The original data of the actual utilization of the FDI were in units of USD. Therefore, this article made a conversion based on the intermediate exchange rate of each year. In addition, the calculation formula of per capita education level was as follows:

(3) $$Education_{i,t}=\left(x_{i,1}\times 0+x_{i,2}\times 6+x_{i,3}\times 9+x_{i,4}\times 12+x_{i,5}\times 16\right)/X$$

where $x_{i,1}$, $x_{i,2}$, $x_{i,3}$, $x_{i,4}$ and $x_{i,5}$ represent the number of employed people with illiteracy, primary school, junior high school, senior high school, junior college, and above education level in province *i* in each year, respectively. The length of schooling is set as 0, 6, 9, 12 and 16 years, respectively, and X is the total number of employed people. In China’s socialist market economy, improving the marketization level is an important driving force behind social progress. Therefore, this article referred to the “general index of marketization” of each province, as compiled by Wang et al., to indicate the degree of marketization [34]. In addition, this article also added the proportion of the added value of the secondary industry in GDP, the number of patent applications per 10,000 people, and the growth rate of the total population of the region relative to the previous year to express the industrial structure, technological innovation level, and population growth rate, respectively. This method was used to control the impact of industrial structure upgrading, technological innovation, and population growth on food safety [35,36]. The above data were from the China Statistical Yearbook, China Economic Database (CEIC), and China Stock Market & Accounting Research Database (CSMAR). In addition, some variables were treated by logarithm. Table 2 shows the statistical results of related variables.

## 3. Results

### 3.1. Spatial Autocorrelation Test

A spatial econometric model is based on the spatial correlation between sample data. Therefore, a spatial autocorrelation test of the sample data is needed first. Based on the exploratory spatial data analysis (ESDA) method, this research investigates the global and local spatial correlations of different spatial unit observations of related variables. The following are the formulas used to calculate the global Moran’s I index.

(4) $$I=\frac{n\sum_{i=1}^{n}\sum_{j=1}^{n}W_{i,j}\left(x_{i}-\bar{x}\right)\left(x_{j}-\bar{x}\right)}{\sum_{i=1}^{n}\sum_{j=1}^{n}W_{i,j}\sum_{i=1}^{n}{\left(x_{i}-\bar{x}\right)}^{2}}=\frac{\sum_{i=1}^{n}\sum_{j=1}^{n}W_{i,j}\left(x_{i}-\bar{x}\right)\left(x_{j}-\bar{x}\right)}{S^{2}\sum_{i=1}^{n}\sum_{j=1}^{n}W_{i,j}}$$

where $x_{i}$ and $x_{j}$ are the measurement of the food safety performance in provinces i and j; $W_{i,j}$ indicates the weight of provinces *i* and *j*; n is the number of observations;$S^{2}$ is the variance; and $\bar{x}$ is the mean value. The index values range from −1 to 1. If I > 0, this means that a positive spatial autocorrelation exists between variables. If I< 0, this means a negative spatial autocorrelation. If I = 0, this means that no spatial autocorrelation exists.

Table 3 indicates that the Moran’s I indexes are significantly positive for the 2005–2015 period. This finding shows that there is a global spatial autocorrelation in food safety performance among the provinces. In addition, there is an increasing trend of the spatial agglomeration of FSP. 

**Figure 2:**
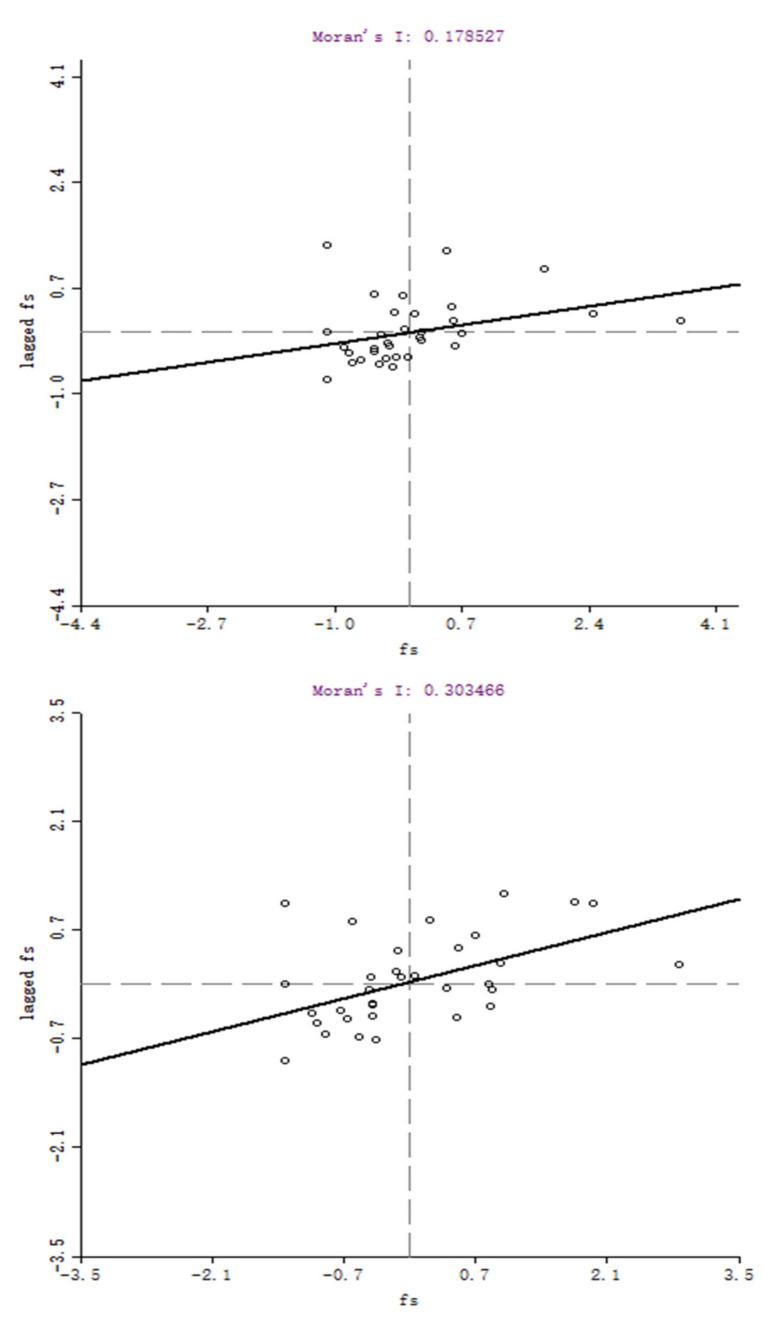
MSP for Chinese provincial FSP based on Ad-weight in 2005 and 2015.

**Figure 3:**
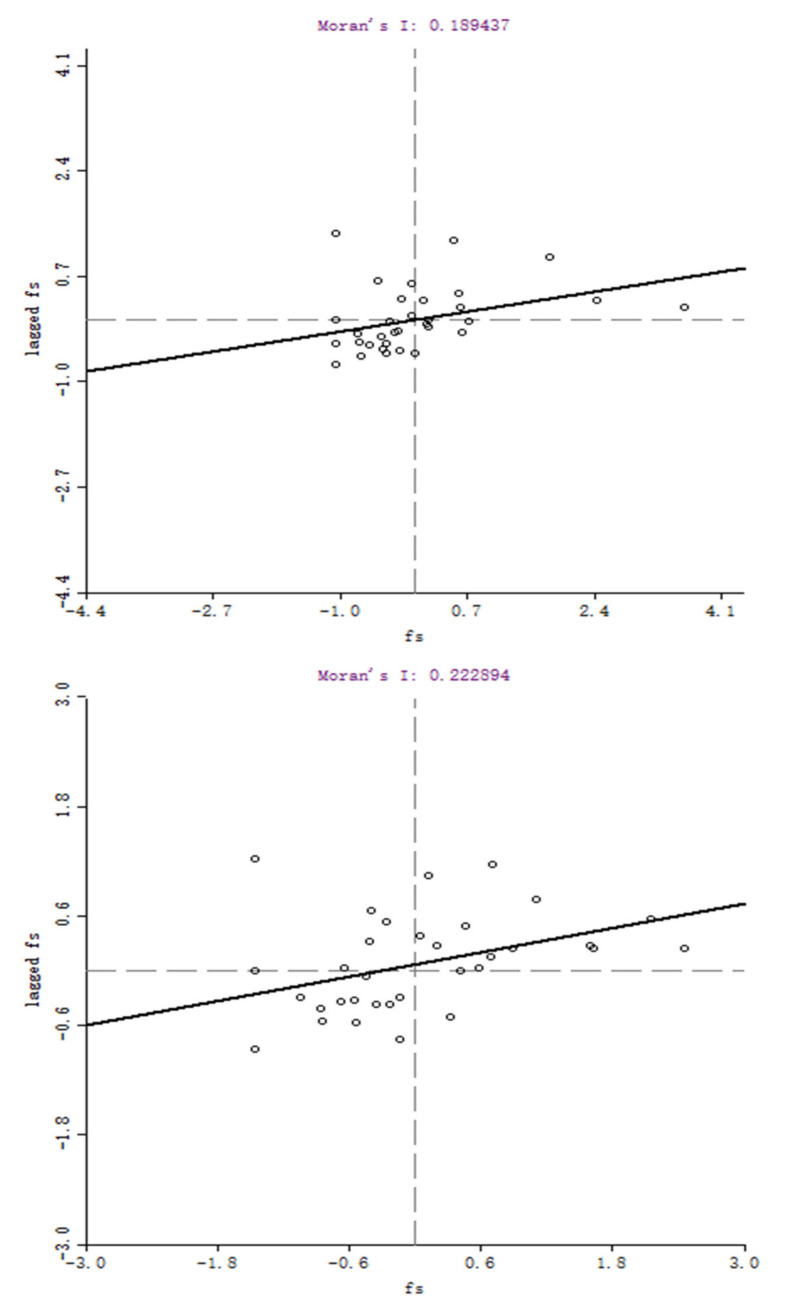
MSP for Chinese provincial FSP based on Geo-weight in 2005 and 2015.

The local spatial autocorrelations are examined through the Moran scatter plots (MSP) by Geoda software (developed by Dr. Luc Anselin and his team, University of Chicago, Chicago, IL, USA). Figure 2 and Figure 3, respectively, report the MSP of food safety performance in 2005 and 2015, based on Ad-weight and Geo-weight. The solid line’s slope represents the Moran’s I global test statistic. The provincial FSP, after standardization, and the spatial lag in FSP are represented by the abscissa and ordinate, respectively. There are four quadrants in the charts. Quadrants one and three show the positive spatial correlation of the observations, while Quadrants two and four are the opposite.

The plots indicate that most observations were located in Quadrants one and three. It can be concluded that the spatial agglomeration by FSP was evident. From 2005 to 2015, the distribution of observations tended to Quadrants one and three, showing that the feature of spatial clustering of FSP had enhanced with time.

### 3.2. Effects of Government Intervention on FSP

#### 3.2.1. OLS Fixed Effects Specification

According to the above-designed Equation (1), a regression estimation is performed based on the OLS method. The control variables are added gradually from Column (1) to Column (5), and the upper and lower 1% winsorizing is adopted. The results are presented in Table 4. The influence coefficient of government intervention on FSP is negative and significant at the level of 1%. This indicates that there is a negative association between government intervention and food safety performance.

As regards the control variables, it is noteworthy that economic development level, degree of marketization, technological innovation, and education level are positively associated with food safety. Moreover, the impact coefficients of openness degree and population growth rate on food safety are significantly negative. This shows that openness has restricted the improvement of food safety through the introduction of foreign capital and the strengthening of international competition. In addition, population growth has exacerbated the dual problems of supervision and the slow improvement of food safety. In addition, the influence coefficients of industrial structure and urbanization level on food safety are not significant. This finding shows that regional industrial structure and urbanization level had no significant association with food safety during the sample inspection period.

#### 3.2.2. Spatial Econometric Specification

Next, this study takes local government competition into consideration and conducts a spatial econometric analysis. Firstly, LR statistics are constructed to test whether the SDM can be simplified into a spatial lag model (SLM) and spatial error model (SEM). According to the results, the SDM should be selected for estimation under Ad-weight and Geo-weight. Then, FE and RE are selected according to the Hausman test and AIC principle. The fixed effect for estimation should be chosen based on the results.

Table 5 indicates the results of the SDM estimation of Equation (2). The estimated coefficient of government intervention is significantly negative. This finding shows that there is a negative association between government intervention and local food safety performance when local government competition is considered. The coefficient of the food safety lag term is also significantly negative, indicating that government intervention also has negative space spillovers on food safety in neighboring areas. Therefore, when considering local government competition, the associations between government intervention and food safety show the strategic interaction of the “race to the bottom”.

#### 3.2.3. Spatial Spillover Effect Analysis

This research also analyzes the spatial spillover effect of government intervention on food safety performance. Referring to the research method of Lesage and Fischer [30], this study uses the partial differential method of spatial regression model to decompose the spatial spillover effect into direct effect, indirect effect, and total effect. The results shown in Table 6 explicitly imply that the total, direct, and indirect effects of government intervention on food safety are significantly negative. This shows that government intervention is not only negatively associated with the improvement of food safety in this region, but, also, is negatively associated with the food safety in neighboring areas.

In the estimation that does not consider local government competition (that is, does not consider spatial factors), the spatial spillover effect is set to 0; the estimated coefficient of government intervention on food safety is −0.951. After considering local government competition, the direct effects of government intervention on food safety are −0.747 and −1.238 under Ad-weight and Geo-weight, respectively. By comparing the above two situations, one can find that the negative associations between government intervention and food safety are underestimated when spatial factors are not considered. When local government competition is not considered, local governments will adopt intervention strategies tailored to their local factor endowments and development situations, thereby reducing the effect of competition. After considering local government competition, based on target assessment and promotion incentives (that is, due to the spatial spillover effect), various regions will inevitably compete with each other. This compels local governments to increase their levels of intervention, adopting the same, or even transcending, the strategies as used in neighboring provinces in order to maximize their interests. Finally, there is a “race to the bottom” strategic interaction among governments.

In addition, it can be found from Columns (1) and (3) of Table 5, and Columns (1) and (4) of Table 6, that the direct effects of government intervention on food safety are not consistent with their coefficients in the estimation of SDM. The difference between them reflects the magnitude of the spatial feedback effect. The spatial feedback effect represents the impact of changes in government intervention on the FSP of neighboring regions, and then the feedback impact on the FSP of the region itself. Specifically, under Ad-weight, the direct effect of government intervention on FSP is −0.874; the estimated coefficient is −0.747; and the feedback effect of government intervention is −0.127. Under Geo-weight, the direct effect of government intervention on food safety performance is −1.125; the estimated coefficient is −1.238; and the feedback effect of government intervention is −0.113. These findings show that the feedback effect of government intervention on food safety performance is negative.

### 3.3. Robustness Tests

#### 3.3.1. Variable Displacement Effects

In this research, the ratio of local government fiscal expenditure on general public services to GDP (Govern2), and the ratio of local government public health expenditures to GDP (Govern3), are used as alternatives to the core explanatory variable. The relevant data come from the CEIC. The Hausman test is employed to select both the FE and the RE. As can be concluded from the results, the former should choose the fixed effect model to estimate, while the latter should choose the random effect model to estimate under Ad-weight. The fixed effect model should be chosen to estimate under Geo-weight. The results are shown in Columns (1) to (4) of Table 7. The estimated coefficients of Govern2 and Govern3 are significantly negative. This finding shows that government intervention is negatively associated with FSP, and the above estimation results are robust.

#### 3.3.2. Weight Matrix Displacement Effects

Since the strategic interaction between regions will not only consider neighboring provinces but, also, provinces with similar economic development levels, this article also establishes an economic distance weight matrix (Eco-weight). The matrix element is the reciprocal of the average difference of per capita GDP between each of the two provinces in the sample period. Based on Eco-weight, the Hausman test shows that the FE model should be selected for estimation. The results are presented in Column (5) of Table 7; the results are basically the same as shown in the preceding part of the text. Government intervention is negatively associated with FSP, and, also, has a negative spatial spillover to other provinces.

#### 3.3.3. Endogeneity Analysis

In this research, the SDM is used to solve endogeneity problems caused by measurement errors and missing variables. To test the robustness of endogenous problem solving, a spatial dynamic panel model is built. The model is based on the system generalized method of moments (SYS-GMM) method for re-estimation. Without introducing external instrumental variables (IV), SYS-GMM can select appropriate IV according to the time trend of variables, so as to solve the endogenous problem. The models have passed the Sargan test and Arellano–Bond test, indicating that both the model and the IV are reasonable and effective, and there is no over-identification problem. The results are shown in Column (6) of Table 7. As can be seen, the coefficient of government intervention is significantly positive at the level of 1%, which is consistent with the above estimated results.

### 3.4. Asymmetric Response Analysis

The estimated coefficients of ρ in Table 4 are significantly positive, which is opposite to the coefficient sign of government intervention and its lag term. Therefore, it is necessary to speculate that there is a certain “competitive effect” between the FSP in different provinces. Therefore, this paper uses Fredriksson and Millimet [37] as a reference to explore the correlation between FSP in different provinces by constructing an asymmetric response model based on SYS-GMM. The model is set as follows:

(5) $$Perform_{i,t}=\varphi_{0}+\varphi_{1}I_{i,t}\sum_{n\neq i}W_{i,n,t}Perform_{n,t}+\varphi_{2}\left(1-I_{i,t}\right)\sum_{n\neq i}W_{i,n,t}Perform_{n,t}+\sum \beta_{j}X_{i,t}+\gamma_{t}+\mu_{i}+\varepsilon_{i,t}$$

where $Perform_{i,t}$ and $X_{i,t}$ are the same as in Equation (1); $W_{i,n,t}$ is the corresponding element of the spatial weight matrix; $\sum_{n\neq i}W_{i,n,t}Perform_{n,t}$ is the average value of the FSP of other provinces, except province i, weighted by the spatial weight matrix; $I_{i,t}$ is a display variable. When the value of $Perform_{i,t}\rangle \sum_{n\neq i}W_{i,n,t}Perform_{n,t}$, $I_{i,t}$ is 1, the coefficient $\varphi_{1}$ (Race_bottom) measures the tendency to race to the bottom. Otherwise, $I_{i,t}$ is equal to 0, and the coefficient $\varphi_{2}$ (Race_top) measures the tendency to race to the top. That is to say, if the weighted average of the FSP of a province’s neighbors is currently below that province’s own FSP, the strategic interaction effect is given by $\varphi_{1}$; otherwise, the effect is given by $\varphi_{2}$. If the two tendencies exist simultaneously, the coefficient is compared in order to determine which effect is dominant. When $\varphi_{1}<\varphi_{2}$, this means that the propensity between provinces to race to the top is greater than the propensity to race to the bottom. In addition, the overall performance is the spatial spillover effect of, “If you are high, I will be higher.” When $\varphi_{1}>\varphi_{2}$, the opposite will hold.

Specifically, according to Columns (1) and (2) in Table 8, regardless of whether or not the control variable is added, the coefficients of Race_bottom and Race_top are significantly positive based on Ad-weight. The Race_top is also significantly larger than the Race_bottom, showing stronger behavioral characteristics of racing to the top. According to Columns (3) and (4) in Table 8, regardless of whether or not the control variable is added, the coefficient of Race_bottom is not significant, but Race_top is significantly positive based on Geo-weight, similarly showing stronger behavioral characteristics of racing to the top. What is more, according to Columns (5) and (6) in Table 8, regardless of whether or not the control variable is added, the coefficients of Race_bottom and Race_top are significantly positive based on Eco-weight. The Race_top coefficient is significantly smaller than Race_bottom, showing stronger behavioral characteristics of racing to the bottom. The above results show that, if geographically similar regions are selected as reference objects, the FSP of each province will have a stronger tendency to compete for the better. Also, if regions with similar economic development levels are selected as reference objects, the FSP will have a stronger tendency to compete for the worse.

The positive incentive for FSP is effectively embodied as a “reputation effect”; for example, the demonstration of China’s food safety cities and positive reports from the news media. In addition, the positive incentive also manifests as a kind of “peer effect” that competes with, and promotes, each other. The negative incentive is the need for more cost investment, the “risk effect” of supervision, and the fluke of being exempted from responsibility. Specifically, on the one hand, when geographically similar regions are taken as the reference objects, those regions’ food production, transportation, sales, and supervision are highly correlated. As such, the “yardstick competition” tendency is obviously greater than the “race to the bottom” tendency. On the other hand, when regions with similar economic development levels are taken as the reference objects, if they are among the more developed regions, the fluke mentality that they have already taken the lead will aggravate the motivation to compete for the worse. Owing to the distances involved, the connection between provinces is relatively sparse. Thus, motivation to compete for the better is relatively weak and is obviously less than motivation to compete for the worse. If the competition is between economically underdeveloped regions, the indicators of competition between the regions are generally manifested in the economic growth rate and the scale of investment promotion, while less attention is paid to food safety.

## 4. Discussion and Implications

### 4.1. Discussion

**Figure 4:**
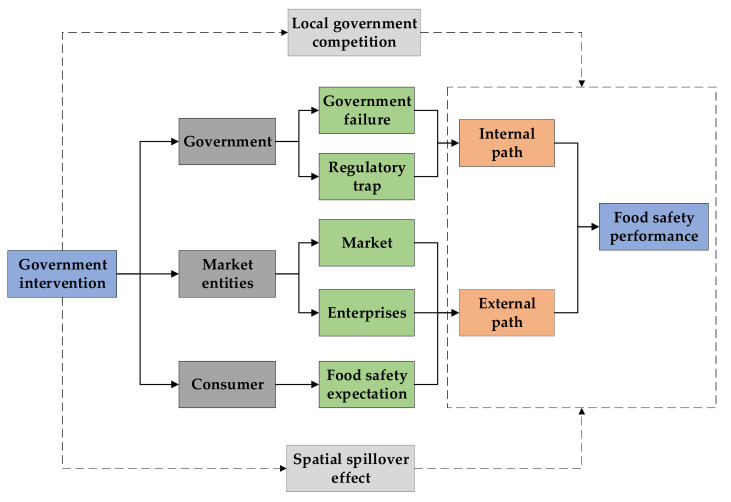
Mechanism path of government intervention on FSP.

Food safety issues usually stem from the asymmetry of information between consumers and suppliers, particularly with regard to the specific attributes of products, the asymmetry of costs and benefits, and the game of interest between related entities [27]. In reality, government intervention is a condition that is necessary to encourage the formation of industry norms [38]. Government intervention is also an important force that drives enterprises to improve food safety [39]. However, there are obstacles in the process of implementing government intervention. As a result of these obstacles, in many cases, government intervention does not achieve the expected goal [40,41]. This study finds that government intervention in China is negatively associated with food safety performance, which is contrary to the research conclusions of Lv et al., Wang et al., and Duan et al., all of which find that government intervention has a positive influence on other industries [42,43,44]. The possible explanations are as follows (Figure 4 presents the mechanism path of government intervention on FSP):

From the government perspective, because a “regulatory trap” [45,46] exists, only when certain constraints are met can the government achieve the expected goal of regulation. On the one hand, although moderate government intervention can promote economic growth [47], due to the limitation of the levels of economic and social development, government intervention capacity is limited. These limitations lead to a gradual increase of food safety problems in the process of continually developing the food industry. On the other hand, the government also has the risk of failure, due to the government’s own limitations [11]. The overlapping functions of government departments, the coexistence of repeated supervision in some cases, the absence of supervision in others, the limited regulatory capacity of the government, and the profit-seeking behavior of officials (and other causes) will all lead to government failure. Due to competition among local governments, the government generally adopts fiscal subsidies and preferential tax policies for the development of the food industry. These measures have led to government rent-seeking behavior and serious resource misallocation [48]. With the continuous development of the market, regulatory capture has made the government unable to strictly supervise, which has inhibited the effectiveness of food safety supervision.

From the perspective of market entities, government intervention may restrict the enthusiasm and initiative of relevant market entities to make the necessary efforts to improve food safety. On the one hand, government intervention highlights the functions of the government, but hinders the role of the market in resource allocation [49]. Corruption and inefficiency are inevitable when a multilevel government intervenes, which may restrict the market-oriented development of food safety supervision. On the other hand, government intervention can reduce the innovation-inducing effect of enterprises, which has a negative influence on the development of enterprises [50]. Although government regulation can increase enterprise investment, those regulations also reduce investment efficiency [15,51]. Affected by cost, lag effect, and externality, food production enterprises may be reluctant to invest in food safety technologies [52], thus further reducing enterprises’ motivation to improve food safety.

From the consumer perspective, strengthening food safety supervision will lead to unrealistically high consumer expectations. This is not conducive to food safety governance [53,54]. When the food safety situation improves, the food safety expectations of the public will be further raised. Once a food incident occurs, however, the public’s trust in the governance capabilities of the government will significantly reduce and fall into a low trust trap. Therefore, government regulation of the food industry will change consumers’ expectations with regard to food safety [55]. Thus, such regulation may have a negative impact on food trading behavior.

According to Waldo Tobler’s first law of geography, “Any geographical thing or attribute has a certain correlation in space, and the closer the distance is, the closer is the relationship” [56]. In this study this same law also applies to government intervention and FSP. After considering local government competition, this paper finds that government intervention has a negative spatial spillover effect on food safety, a conclusion which is similar to that of Shen et al. [57]. Based on the view of local government competition, Shen’s research found that government intervention has significantly exacerbated coastal water pollution. The alternative reasons for the negative spatial effect in this research are as follows: In the context of China’s fiscal decentralization and promotion incentives offered to officials, competition in various aspects undoubtedly exists among local governments, including in areas such as investment and social performance. Owing to the existence of competition, government intervention will also have an association with food safety in neighboring areas. This occurs through spatial spillover effects. Government intervention measures will force neighboring regions to increase government intervention, specifically due to the competitive situation, resulting in a negative association with food safety in neighboring regions. Such a “vicious circle” continues to spread and, eventually, various regions adopt the strategy of “race to the bottom”.

### 4.2. Practical Implications

This study finds that China’s food safety interests still face severe challenges. On the basis of an empirical analysis and discussion, this research puts forward some feasible suggestions regarding how to promote the improvement of food safety.

Firstly, promote the reform of government regulation systems and enhance the effectiveness of government intervention. The spatial econometric estimation results show that there is a significantly negative association between government intervention and food safety. Therefore, on the one hand, an absolute requirement exists to improve food safety supervision and governance mechanisms, reasonably formulate food safety assurance plans, and clarify the division of responsibilities for supervisors. On the other hand, there is a need to establish the long-term guarantees mechanism of food safety, and to speed up the systems construction of food supply chain traceability and integrity.

Secondly, strengthen regional cooperation and the exchange of experiences. Furthermore, improve the joint prevention and control mechanisms of food safety. The results of the asymmetric response analysis show that the FSP in neighboring provinces creates the interaction strategy of “yardstick competition”. Therefore, all regions are supposed to break through geographical boundaries; regional food safety joint guarantee organizations should be established to strengthen regional cooperation. In addition, food safety incidents should be increasingly exposed, with the help of the media; punishments for profit seeking behaviors that endanger food safety should be increased, and propaganda campaigns regarding food safety laws, regulations, and standards should be conducted.

Thirdly, promote the participation of multiple entities in food safety maintenance, and promote the diversification of governance modes. Ensuring food safety is a systematic and complex social project, one which requires not only government intervention but also the cooperation of social forces. Governments should reasonably adjust their terms of reference so as to truly stimulate all market players’ sense of ownership. A government should not only pay attention to the supervision of food safety but should also ensure the effectiveness of the multidirectional information flow between different governance entities [58]. The responsibility of a market is to give full play to that market’s decisive role in resource allocation, in order to achieve the optimal allocation of elements. In addition, food companies must enhance their own sense of social responsibility and promote industrial upgrading and development through technological innovation. Society is an important force behind ensuring food safety. This is especially true of social organizations and industry associations that have multiple roles as participants and promoters of food safety supervision. In addition, society must participate in governance throughout the process and give full play to the relevant roles.

## 5. Conclusions

The results of this study show that, when local government competition is not considered, there is a negative association between government intervention and food safety performance; however, the degree of association is underestimated. This also confirms, to a certain extent, the existence of a “regulatory trap”. When local government competition is considered, government intervention not only has a negative association with food safety in the region, but, also, has a negative spatial spillover to neighboring regions, creating and showing the strategic characteristics of a “race to the bottom” strategy. After the decomposition of the spatial effect, it is found that the total effect, direct effect, and indirect effect of government intervention on food safety are significantly negative; there is also a negative feedback effect on food safety. Through the analysis of an asymmetric response model, it is found that, if geographically similar regions are selected as the reference objects, the food safety performance of each province has a stronger tendency to compete for the better. If the regions with similar economic development level are selected as the reference object, there is a stronger tendency for food safety performance to compete for the worse.

Nevertheless, despite this paper’s meaningful findings, this research has some limitations, which could, in turn, be used as reference points for future studies. On the one hand, although this study successfully estimates the associations between government intervention and food safety performance in China, due to the imperfect (or missing) statistics on food-related data, the indicators selected in this study are representative but are not perfect. In follow-up work, further improvements can be made in terms of data collection and integration. On the other hand, the analysis in this study is aimed at China, a special developing country. Considering the differences of government intervention and food safety regulation in countries with different economic and cultural attributes, future studies could be conducted in other countries.

## Figures and Tables

**Table 1:** Primary variables and definitions.

Variable Type	Symbol	Variable Name	Definition	Reference
Dependent variable	FSP	Food safety performance	1/number of food safety incidents	Zhang et al. [31]
Independent variable	Govern	Government intervention	General budget expenditure of local finance×(output value of national food industry/GDP)/regional GDP	Shi and Shen [32]
Control variable	Pergdp	Economic development level	Regional GDP/total population	Cheng et al., Wang et al., Yu et al., and Huang et al. [33,34,35,36]
	Open	Openness degree	(The actual utilization of FDI in the region/regional GDP) × 100%	
	Market	Degree of marketization	Refer to the “general index of marketization” of each province, compiled by Wang et al.	
	Industry	Industrial structure	(Added value of secondary industry in the region/regional GDP) × 100%	
	Population	Population growth rate	[(Total population at the end of current year/total population at the end of last year) − 1] × 100%	
	Innovate	Technological innovation	(Number of regional patent applications authorized/total population) × 10,000	
	Urbanization	Urbanization level	(Urban population/total population) × 100%	
	Education	Education level	(Number of primary school graduates × 6 + number of junior high school graduates × 9 + number of senior high school graduates × 12 + number of junior college or above graduates × 16)/total population	

**Table 2:** Summary statistics.

Variable Type	Symbol	Sample Size	Mean	Standard Deviation	Min	Max
Dependent variable	FSP	330	0.2766	0.2797	0.0240	1.6393
Independent variable	Govern	330	0.2107	0.0825	0.0719	0.6357
Control variable	Pergdp	330	10.2740	0.6272	8.5277	11.5895
	Open	330	0.0557	0.0737	0.0071	0.7503
	Market	330	1.9743	0.2580	1.1694	2.5424
	Industry	330	0.4760	0.0784	0.1974	0.6150
	Population	330	5.2561	2.6170	−0.6000	11.7800
	Innovate	330	1.5594	0.9506	0.2554	4.2080
	Urbanization	330	0.5174	0.1413	0.2687	0.8960
	Education	330	2.2595	0.0999	1.9985	2.5762

**Table 3:** Morans’I index of food safety performance.

Year	Ad-Weight		Geo-Weight	
	Moran’s I Index	*p*-Value	Moran’s I Index	*p*-Value
2005	0.178	0.000	0.189	0.000
2006	0.174	0.000	0.157	0.000
2007	0.289	0.001	0.098	0.002
2008	0.245	0.006	0.047	0.005
2009	0.269	0.005	0.046	0.007
2010	0.278	0.000	0.125	0.004
2011	0.314	0.000	0.137	0.014
2012	0.201	0.017	0.159	0.048
2013	0.218	0.008	0.182	0.030
2014	0.282	0.002	0.186	0.013
2015	0.303	0.001	0.222	0.019

**Table 4:** Estimation results of OLS fixed effect model.

Variable	FSP	FSP	FSP	FSP	FSP
	(1)	(2)	(3)	(4)	(5)
Govern	−1.389 ***	−1.193 ***	−1.171 ***	−1.039 ***	−0.951 ***
	(0.428)	(0.416)	(0.256)	(0.298)	(0.215)
Pergdp		0.282 ***	0.272 ***	0.286 ***	0.278 **
		(0.079)	(0.102)	(0.105)	(0.124)
Open		−0.111 ***	−0.105 ***	−0.050 **	−0.039 *
		(0.034)	(0.020)	(0.024)	(0.021)
Market			0.075 ***	0.194 **	0.199 **
			(0.017)	(0.091)	(0.081)
Industry			−0.010	−0.071	−0.056
			(0.306)	(0.304)	(0.303)
Population				−0.021 *	−0.018 **
				(0.012)	(0.007)
Innovation				0.116 ***	0.134 ***
				(0.043)	(0.046)
Urbanization					0.482
					(0.607)
Education					0.935 **
					(0.443)
Regional fixed effect	yes	yes	yes	yes	yes
Year fixed effect	yes	yes	yes	yes	yes
_cons	0.961 ***	3.613 ***	3.690 ***	3.851 ***	5.590 ***
	(0.051)	(0.743)	(0.891)	(0.946)	(1.252)
observations	330	330	330	330	330
Adj $R^{2}$	0.663	0.676	0.674	0.686	0.689

Notes: The robust standard errors are reported in parentheses; ***, ** and * represent significance at the levels of 1%, 5% and 10%, respectively. The heading “Observations” represents the number of samples.

**Table 5:** Estimation results of SDM model.

Variable	Ad-Weight		Geo-Weight	
	FE	SE	FE	SE
	(1)	(2)	(3)	(4)
Govern	−0.615 ***	−0.747 **	−1.238 ***	−1.190 ***
	(0.158)	(0.326)	(0.314)	(0.282)
Pergdp	0.383 ***	0.400 ***	0.306 **	0.297 ***
	(0.122)	(0.112)	(0.122)	(0.107)
Open	−0.080	−0.099	0.120 ***	−0.085 ***
	(0.136)	(0.148)	(0.036)	(0.021)
Market	0.299 **	0.293 **	0.331 **	0.368 ***
	(0.127)	(0.130)	(0.129)	(0.133)
Industry	0.102	0.086	−0.008	−0.032
	(0.281)	(0.222)	(0.323)	(0.237)
Population	0.004	0.013	0.078 **	0.025 ***
	(0.013)	(0.011)	(0.036)	(0.010)
Innovation	0.126 ***	0.068 *	0.126 ***	0.104 ***
	(0.042)	(0.040)	(0.044)	(0.040)
Urbanization	0.561 **	1.193 ***	0.544	0.681
	(0.243)	(0.406)	(0.650)	(0.428)
Education	0.926 **	0.742**	0.838 *	0.844 **
	(0.396)	(0.351)	(0.433)	(0.371)
W × Govern	−0.822 **	−0.676 **	−7.723 ***	−1.257 ***
	(0.326)	(0.312)	(2.816)	(0.348)
W × Pergdp	0.642 ***	0.257 *	1.308	0.227
	(0.232)	(0.156)	(0.853)	(0.270)
W × Open	1.245	−1.747	−3.529	−3.141
	(1.591)	(1.213)	(4.010)	(2.166)
W × Market	0.381 ***	0.494 ***	2.345 **	0.438 **
	(0.089)	(0.159)	(1.118)	(0.180)
W × Industry	−0.927	−0.664	−2.146	−0.698
	(0.615)	(0.436)	(2.425)	(1.064)
W × Population	−0.048 *	−0.009 *	−0.165 **	−0.044 *
	(0.025)	(0.004)	(0.073)	(0.023)
W × Innovation	0.030	−0.061	−0.173	0.031
	(0.084)	(0.061)	(0.372)	(0.173)
W × Urbanization	−1.009	−0.908	2.298	−1.790
	(1.399)	(0.812)	(4.118)	(1.573)
W × Education	0.802 **	0.992 **	0.175 ***	1.310 **
	(0.339)	(0.481)	(0.035)	(0.666)
ρ	0.260 ***	0.639 ***	0.265 ***	0.703 ***
	(0.070)	(0.044)	(0.083)	(0.069)
Log−likelihood	275.926	171.307	269.717	177.948
LR_spatial_lag	12.932 **	33.082 ***	18.415 **	31.552 ***
LR_spatial_error	15.319 ***	23.366 ***	19.751 ***	20.70 *
AIC	−457.853	−244.614	−445.436	−257.897
Hausman_test	118.974 ***		406.425 ***	
Observations	330	330	330	330
R2	0.090	0.289	0.335	0.418

Notes: The robust standard errors are reported in parentheses; ***, ** and * represent significance at the levels of 1%, 5% and 10%, respectively. The heading “Observations” represents the number of samples.

**Table 6:** Decomposition results of spatial effect.

Variable	Ad-Weight			Geo-Weight		
	Direct	Indirect	Total	Direct	Indirect	Total
	(1)	(2)	(3)	(4)	(5)	(6)
Govern	−0.874 **	−0.306 **	−0.949 ***	−1.125 ***	−8.196 **	−11.305 ***
	(0.361)	(0.142)	(0.154)	(0.381)	(3.794)	(2.986)
Pergdp	0.402 ***	0.018 **	0.384 **	0.305 **	1.405	1.100 **
	(0.103)	(0.008)	(0.174)	(0.109)	(1.000)	(0.451)
Open	−0.489	−4.648	−5.137	0.154 ***	−4.133	−4.288 ***
	(0.407)	(3.111)	(3.364)	(0.053)	(3.387)	(0.855)
Market	0.224 *	0.788 ***	0.564 *	0.343 ***	2.697 *	3.040 **
	(0.120)	(0.290)	(0.292)	(0.130)	(1.430)	(1.482)
Industry	−0.048	−1.620	−1.669	−0.006	−2.179	−2.185
	(0.244)	(1.138)	(1.281)	(0.314)	(2.802)	(2.970)
Population	0.018	−0.043 **	−0.061 **	0.010 *	−0.184 *	−0.194 **
	(0.011)	(0.020)	(0.025)	(0.005)	(0.094)	(0.095)
Innovation	0.065 **	−0.040	0.025 *	0.126 ***	−0.156	0.031 ***
	(0.029)	(0.145)	(0.014)	(0.048)	(0.447)	(0.009)
Urbanization	1.147 ***	−0.526	0.621 ***	0.544 **	2.862	3.407 **
	(0.424)	(2.021)	(0.145)	(0.248)	(4.811)	(1.341)
Education	0.598 *	1.254 **	1.655 *	0.802 *	0.650 **	1.452 **
	(0.359)	(0.501)	(0.354)	(0.440)	(0.270)	(0.610)
Observations	330	330	330	330	330	330

Notes: The robust standard errors are reported in parentheses; ***, ** and * represent significance at the levels of 1%, 5% and 10%, respectively. The heading “Observations” represents the number of samples.

**Table 7:** Results of robustness tests.

Variable	Weighting Scheme					
	Ad-Weight	Geo-Weight	Ad-Weight	Geo-Weight	Eco-Weight	
	(1)	(2)	(3)	(4)	(5)	(6)
L.FSP						0.264 ***
						(0.046)
Govern					−2.415 ***	−0.916 ***
					(0.524)	(0.194)
Govern2	−0.112 ***	−0.327 ***				
	(0.041)	(0.090)				
Govern3			−2.012 ***	−1.004 **		
			(0.519)	(0.409)		
Pergdp	0.217	0.058	0.047	0.098	0.313 ***	0.007
	(0.133)	(0.128)	(0.076)	(0.128)	(0.115)	(0.142)
Open	−0.090 ***	−0.234 *	−0.329 **	0.120	−0.002	−0.153
	(0.025)	(0.138)	(0.132)	(0.139)	(0.141)	(0.094)
Market	0.030	0.062	0.068	0.027	−0.151	0.093
	(0.117)	(0.118)	(0.108)	(0.128)	(0.128)	(0.197)
Industry	0.061	−0.213	0.554 ***	−0.163	0.053	−0.098
	(0.298)	(0.353)	(0.186)	(0.360)	(0.285)	(0.396)
Population	−0.024 *	−0.014	−0.006	−0.011	0.015	−0.020
	(0.013)	(0.013)	(0.007)	(0.013)	(0.012)	(0.013)
Innovation	0.013	0.003	0.049*	0.013 ***	0.090 **	0.100 **
	(0.047)	(0.053)	(0.029)	(0.003)	(0.043)	(0.043)
Urbanization	0.600	0.120	0.133	0.330	0.871	0.289
	(0.626)	(0.664)	(0.267)	(0.672)	(0.602)	(0.828)
Education	0.148 ***	0.195 ***	0.207	0.289	0.374	0.650 *
	(0.044)	(0.030)	(0.255)	(0.433)	(0.427)	(0.392)
W × Govern					−1.066 ***	
					(0.321)	
W × Govern2	−0.223 **	−2.950 **				
	(0.092)	(1.392)				
W × Govern3			−1.063 **	−9.282 ***		
			(0.508)	(1.774)		
W × Pergdp	0.692 ***	1.444	−0.032	0.928	0.472	
	(0.262)	(0.892)	(0.136)	(0.841)	(0.306)	
W × Open	−0.181	1.708	−1.504	0.279	−1.161 **	
	(1.598)	(2.079)	(1.029)	(1.967)	(0.487)	
W × Market	0.015	−0.322	0.255 *	−1.247	0.179	
	(0.232)	(1.074)	(0.132)	(1.145)	(0.338)	
W × Industry	−1.595 **	−5.822 **	−0.478	−4.862 *	2.570 ***	
	(0.672)	(2.608)	(0.396)	(2.635)	(0.886)	
W × Population	−0.049 **	−0.105	−0.096 ***	−0.089	0.083 **	
	(0.025)	(0.079)	(0.029)	(0.078)	(0.036)	
W × Innovation	0.043 ***	0.260 ***	0.098 ***	0.371 ***	−0.180	
	(0.007)	(0.077)	(0.023)	(0.086)	(0.148)	
W × Urbanization	−1.746	−1.685	0.195	0.398	−0.177	
	(1.558)	(4.366)	(0.588)	(4.370)	(1.843)	
W × Education	1.115	3.188	0.462	3.374	−0.073	
	(0.943)	(3.489)	(0.407)	(3.469)	(1.019)	
ρ	0.205 **	0.013 ***	0.556 ***	0.712 **	0.456 ***	
	(0.080)	(0.005)	(0.057)	(0.320)	(0.058)	
Log-likelihood	271.727	266.937	196.681	267.287	270.448	
AIC	−449.455	−439.875	−295.363	−440.575	−446.896	
Regional fixed effect	yes	yes	yes	yes	yes	yes
Year fixed effect	yes	yes	yes	yes	yes	yes
AR(2)						0.780
Sargan test						1.000
Observations	330	330	330	330	330	270

Notes: The robust standard errors are reported in parentheses; ***, ** and * represent significance at the levels of 1%, 5% and 10%, respectively. The heading “Observations” represents the number of samples.

**Table 8:** Estimation results of asymmetric response model.

Variable	Weighting Scheme					
	Ad-Weight		Geo-Weight		Eco-Weight	
	(1)	(2)	(3)	(4)	(5)	(6)
L.FSP	0.213 ***	0.193 ***	0.242 ***	0.220 ***	0.254 ***	0.226 ***
	(0.064)	(0.050)	(0.068)	(0.050)	(0.065)	(0.049)
Race_bottom	50.982 **	53.080 **	103.335	96.547	40.714 **	39.924 *
	(22.778)	(24.342)	(72.648)	(65.187)	(19.988)	(21.236)
Race_top	70.530 ***	70.437 ***	136.947 *	128.917 *	12.707 ***	13.050 ***
	(18.186)	(19.471)	(70.677)	(72.416)	(3.529)	(3.085)
Govern	−1.027 ***	−0.927 ***	−1.210 ***	−1.012 ***	−1.611 ***	−1.471 ***
	(0.328)	(0.307)	(0.286)	(0.246)	(0.212)	(0.233)
Pergdp		0.035		0.020		0.035
		(0.117)		(0.121)		(0.143)
Open		−0.134 *		−0.182 *		−0.206 **
		(0.080)		(0.095)		(0.102)
Market		0.085 ***		0.094 ***		0.112 ***
		(0.029)		(0.035)		(0.038)
Industry		−0.123		−0.139		−0.354
		(0.340)		(0.340)		(0.372)
Population		−0.072 ***		−0.088 ***		−0.011
		(0.026)		(0.030)		(0.011)
Innovation		0.051		0.079 ***		0.079 ***
		(0.046)		(0.027)		(0.024)
Urbanization		0.046		−0.122		−0.031
		(0.765)		(0.789)		(0.819)
Education		0.358		0.480 ***		0.625
		(0.450)		(0.144)		(0.386)
Regional fixed effect	yes	yes	yes	yes	yes	yes
Year fixed effect	yes	yes	yes	yes	yes	yes
AR(2)	0.540	0.893	0.439	0.198	0.980	0.761
Sargan test	0.588	1.000	0.368	0.720	0.811	0.470
Observations	270	270	270	270	270	270

Notes: The robust standard errors are reported in parentheses; ***, ** and * represent significance at the levels of 1%, 5% and 10%, respectively. The heading “Observations” represents the number of samples.

## Data Availability

Not applicable. No new data were created or analyzed in this study.
